# Alopecia areata-like presentations with mogamulizumab therapy

**DOI:** 10.1016/j.jdcr.2023.08.044

**Published:** 2023-09-16

**Authors:** Colin M. Kincaid, Ajay N. Sharma, Bonnie A. Lee, Lauren C. Pinter-Brown, Janellen Smith, Kenneth Linden, Natasha A. Mesinkovska

**Affiliations:** a Department of Dermatology, University of California, Irvine, California; b Department of Hematology/Oncology, University of California, Irvine, California

**Keywords:** alopecia, alopecia areata, cutaneous T-cell lymphoma, drug rash, hair loss, mogamulizumab, mycosis fungoides, Sézary syndrome

## Background

Mogamulizumab is an anti- C-C chemokine receptor 4 (CCR4) monoclonal antibody currently approved for the treatment of relapsed or refractory mycosis fungoides (MF) and Sézary syndrome (SS).^1^ While it has been shown to be efficacious for this indication, its use is commonly associated with drug eruptions known as mogamulizumab-associated rashes (MAR).^1^ Recent reports of “alopecia areata” (AA) following treatment raise the question of whether mogamulizumab may also increase the risk of immune-mediated cutaneous adverse events.^2^, ^3^, ^4^ We report a case of a man with SS treated with mogamulizumab who developed MAR and alopecia universalis-like features, and we review the literature and possible mechanisms for mogamulizumab-associated alopecia.

### Report of case

A 71-year-old man presented to the clinic with 2 weeks of widespread alopecia and follicular dermatitis affecting his extremities. His history began 5 years prior when he had sought treatment for intractable pruritus without cutaneous changes (2019). Flow cytometry revealed a CD4+ T-cell lymphoproliferative disorder with cytology suggestive of Sezary cells, however, his lack of skin involvement led to deferral of a skin biopsy and delay in diagnosis. He was treated with 5 mg prednisone PO daily which partially controlled his pruritus. Three years later, he underwent a blind biopsy of healthy appearing skin, revealing atypical lymphoid cells infiltrating hair follicles with a markedly elevated CD4:CD8 ratio and diminished CD7 expression (2022). Additional workup, including repeat blood flow cytometry (43% CD4+/CD26-, 30% CD4+/CD7-) and skin T-cell receptor (TCR) gene rearrangement studies (beta and gamma positive), confirmed a diagnosis of SS without erythroderma, or “invisible” SS, an atypical but reported entity in the literature.^5^

**Fig 1:**
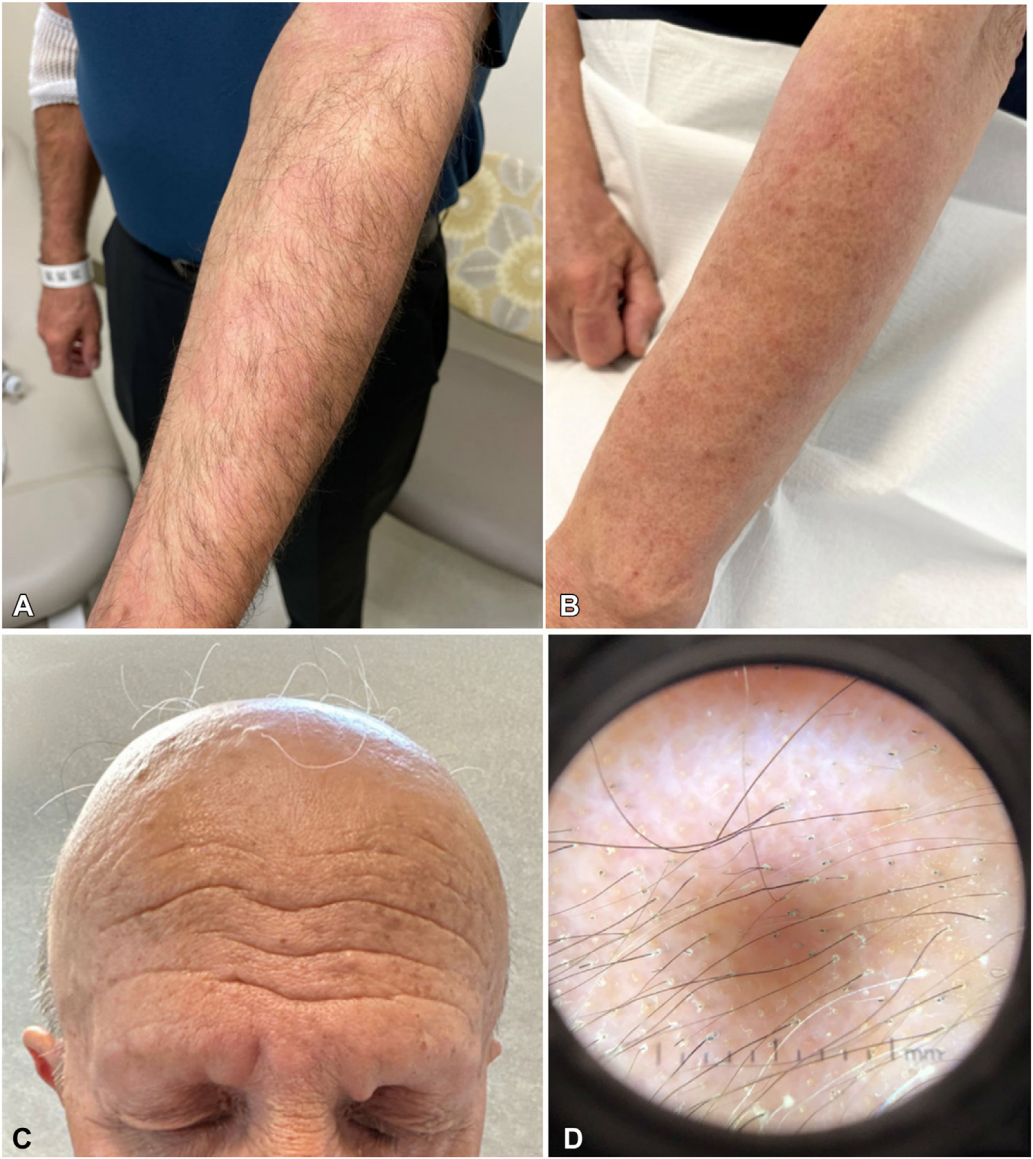
Scaly, faint erythematous plaques on the arm after 2 months of mogamulizumab treatment (**A**). Hair loss and follicular papules after 6 months of treatment (**B**). Alopecia of the scalp, eyebrows, and eyelashes after 6 months of treatment (**C**). Preserved follicular ostia, perifollicular scale, *yellow dots*, and rare exclamation point hairs under dermoscopy (**D**).

Upon diagnosis of SS, he was enrolled into a clinical trial for monthly mogamulizumab therapy (2022). Two months after initiation of therapy, he developed scaly, faintly erythematous plaques on his bilateral arms (Fig 1, *A*). Histopathology demonstrated a superficial lymphocytic infiltrate with spongiosis and a CD4:CD8 ratio of 4:1. A drug eruption was favored and treatment was held. Upon resolution 3 weeks later, treatment cycles were resumed.

**Fig 2:**
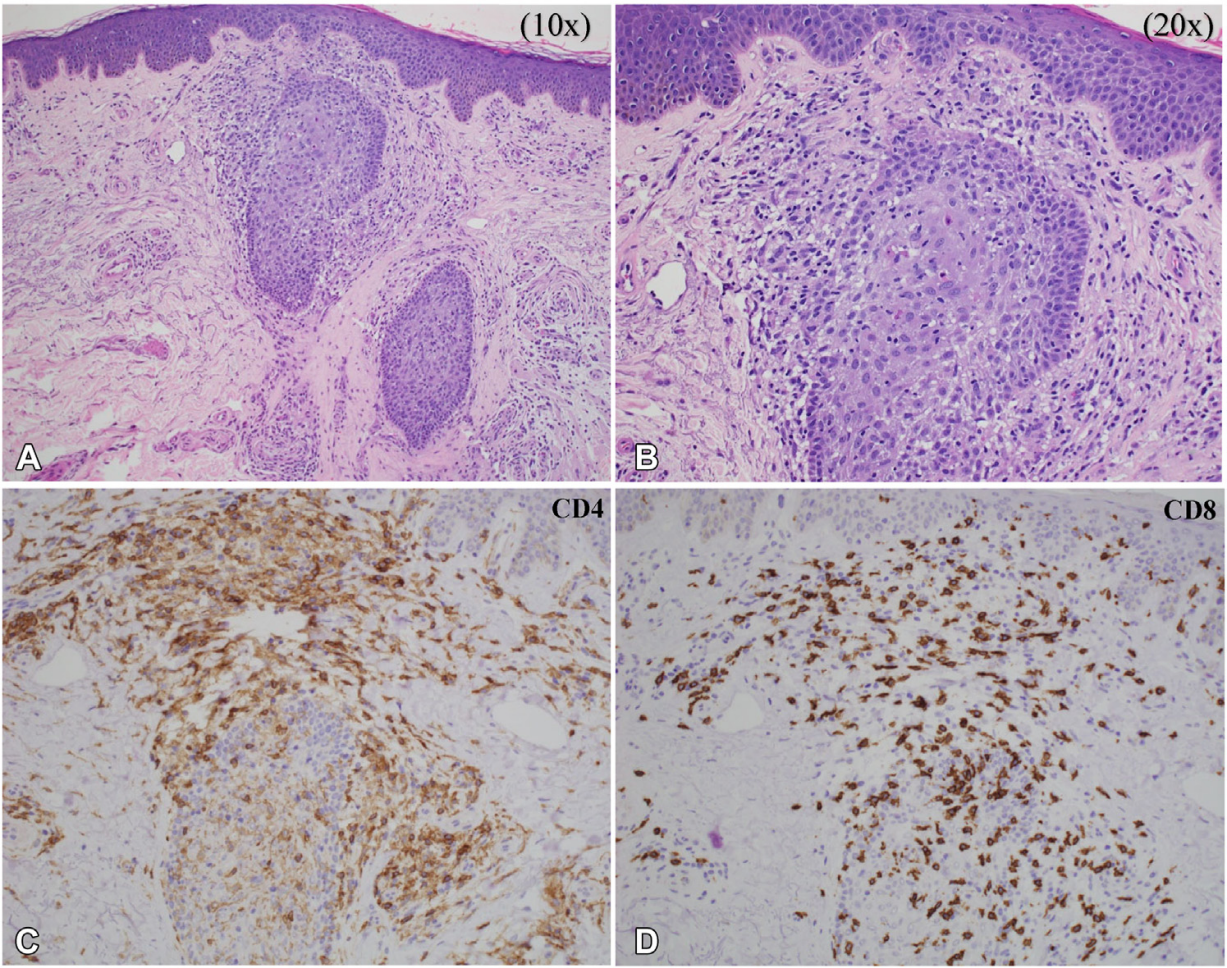
Histopathology of an arm papule (H&E) demonstrating intrafollicular lymphocytes at 10× (**A**), and 20× (**B**), magnification. Immunohistochemistry at 20×: CD4 (**C**), and CD8 (**D**), stains demonstrating a predominance of CD8+ lymphocytes.

At his current presentation 6 months into treatment, he developed widespread alopecia of the frontal and vertex scalp, extremities (Fig 1, *B*), eyebrows (Fig 1, *C*), and trunk (2023). Preserved follicular ostia and rare exclamation point hairs were appreciated under dermoscopy, features suggestive of AA (Fig 1, *D*).^6^ Examination of his arms revealed scaly, hyperpigmented, pinpoint follicular papules. Biopsy of a right forearm follicular papule demonstrated epidermal spongiosis, relatively small intrafollicular CD8+ T-cells, and a lymphohistiocytic infiltrate surrounding follicles in catagen/telogen stage (Fig 2, *A* and B). Immunohistochemistry now demonstrated an inverse CD4:CD8 ratio (approximately 1:6) (Fig 2, *C* and D). Although the histopathologic pattern overlaps with follicular MF, given the morphology of the lymphocytes, and the increasingly frequent reports of CD8+ MF-like lymphocytic infiltrates in patients treated with mogamulizumab, this was favored to be MAR with associated medication-induced alopecia. Treatment was subsequently held for confirmatory testing and interval assessment of the rash without immunotherapy. Alopecia treatment with minoxidil 2.5 mg PO daily and 2.5 mg/cc intralesional triamcinolone to his bilateral eyebrows was initiated. One month after treatment cessation (1 therapeutic cycle), TCR gene rearrangement studies of peripheral blood and skin were negative, flow cytometry showed no abnormal populations, and his rash largely resolved, favoring a diagnosis of MAR with a cutaneous phenotypic switch from CD4 to CD8+ T cells over persistence of the patient’s MF. Now 5 months (six cycles) off mogamulizumab, our patient’s SS remains in remission without any recurrence of MAR.

## Discussion

Cutaneous drug eruptions are one of the most common adverse events associated with mogamulizumab therapy. Although 24% of patients in phase III trials developed a drug rash, the incidence has been reported to be as high as 63%.^1^^,^^7^^,^^8^ MAR are clinically and histopathologically heterogenous and can often mimic residual or recurrent cutaneous T-cell lymphoma.^7^^,^^9^ Given that MARs appear to indicate a favorable clinical response to treatment,^9^ distinguishing them from residual or even progressive disease is imperative. Histologic features that favor MAR in contrast to cutaneous T-cell lymphoma include a normal or inverted CD4:CD8 ratio and polyclonality of TCR genes.^7^^,^^9^

In phase III clinical trials, alopecia was noted to be a common (7%) adverse event associated with mogamulizumab treatment,^8^ though its histopathologic and clinical features are rarely described.^5^ To date, three reports (6 patients total) detail the AA-like presentations following mogamulizumab therapy (Table I).^2^, ^3^, ^4^ The onset of alopecia ranges from 3 to 16 months after treatment initiation, commonly resembling alopecia totalis or universalis. Three reports have described histopathologic features, with one case^2^ demonstrating follicles in telogen phase, and two cases^3^ demonstrating peribulbar CD8+ T-cell infiltration. Acute AA is classically marked by a peribulbar lymphocytic infiltrate.^6^ In contrast, the present case demonstrated a CD8+ predominant lymphocytic infiltration involving the entire follicle.

**Table I:** Summary of alopecia areata-like presentations following mogamulizumab therapy

Source	Age	Sex	Alopecia presentation	Time to alopecia onset	Findings	CTCL response at time of alopecia presentation
Raval et al^2^	42	F	Alopecia Universalis	16 mo	*-* bx: reduced follicle count, follicles in telogen	Complete
Amatore et al∗^,^^3^	58	F	Alopecia Totalis	16 mo	*-* decreased peripheral Tregs	Complete
	*71*	F	Alopecia Totalis	6 mo	*-* decreased peripheral Tregs	Complete
	35	F	Alopecia Totalis	14 mo	NR	Partial
	81	M	Ophiasis	11 mo	NR	Partial
Bonnet et al^4^	61	F	Alopecia Areata	3 mo	*-* decreased peripheral Tregs	Complete
Present case	71	M	Alopecia Universalis	6 mo	*-* bx: intrafollicular CD8+ lymphocytes	Complete

*bx*, Biopsy; *NR*, none reported; *Treg*, regulatory T-cells.

∗ Two biopsies showed peribulbar CD8+ T-cell infiltration, however patients that had biopsies were not specified.

Mogamulizumab achieves its effect by targeting CCR4-expressing Sézary cells, thus inducing antibody-dependent cellular cytotoxicity.^1^ However, CCR4 is also highly expressed by regulatory T-cell (Treg) cells which can thus lead to their off-target depletion during therapy.^1^ This depletion is thought to drive a homeostatic proliferation of CD8+ T-cells, especially in patients who develop MARs.^10^ The resulting skew towards CD8+ T-cell proliferation may underlay the activation of antitumor immunity^4^ as well as the mechanism responsible for AA-like features during therapy. The classic pathogenesis of AA is thought to involve the collapse of hair follicle immune privilege with insufficient Treg activity and intrafollicular infiltration of autoreactive CD8+ T-cells.^6^ In line with this mechanism, reported cases of mogamulizumab-associated alopecia, including the present case, have demonstrated a depletion of peripheral Tregs and/or predominance of CD8+ T-cells on biopsy.

Given the inversion of the CD4:CD8 ratio over the course of mogamulizumab treatment demonstrated in our patient, mogamulizumab-associated alopecia appears to be more akin to MAR rather than true AA. This finding suggests that mogamulizumab-associated alopecia may similarly be a positive prognostic factor. Indeed, the majority of reported cases of mogamulizumab-associated alopecia have demonstrated a positive response to treatment (Table I). As with MARs, the development of mogamulizumab-associated alopecia may be associated with favorable outcomes, but larger studies and longer follow-up periods are needed to confirm this observation.

## Conflicts of interest

None disclosed.

## References

[1] Lewis D.J., Rook A.H. (2020). Mogamulizumab in the treatment of advanced mycosis fungoides and Sézary syndrome: safety and efficacy. Expert Rev Anticancer Ther.

[2] Raval N.S., Alexander N.A., De Monnin K. (2022). Alopecia areata after mogamulizumab treatment. JAAD Case Rep.

[3] Amatore F., Dereure O., Delaporte E., Ram-Wolff C., Bagot M. (2021). Is mogamulizumab-induced alopecia areata associated with favorable outcomes in Sézary syndrome?. Eur J Cancer.

[4] Bonnet P., Battistella M., Roelens M. (2019). Association of autoimmunity and long-term complete remission in patients with Sézary syndrome treated with mogamulizumab. Br J Dermatol.

[5] Deen K., O'Brien B., Wu J. (2015). Invisible mycosis fungoides: not to be missed in chronic pruritus. Dermatol Ther.

[6] Guo H., Cheng Y., Shapiro J., McElwee K. (2015). The role of lymphocytes in the development and treatment of alopecia areata. Expert Rev Clin Immunol.

[7] Hirotsu K.E., Neal T.M., Khodadoust M.S. (2021). Clinical characterization of mogamulizumab-associated rash during treatment of mycosis fungoides or Sézary syndrome. JAMA Dermatol.

[8] Kim Y.H., Bagot M., Pinter-Brown L. (2018). Mogamulizumab versus vorinostat in previously treated cutaneous T-cell lymphoma (MAVORIC): an international, open-label, randomised, controlled phase 3 trial. Lancet Oncol.

[9] Wang J.Y., Hirotsu K.E., Neal T.M. (2020). Histopathologic characterization of mogamulizumab-associated rash. Am J Surg Pathol.

[10] Saito M., Ishii T., Urakawa I. (2020). Robust CD8+ T-cell proliferation and diversification after mogamulizumab in patients with adult T-cell leukemia-lymphoma. Blood Adv.

